# Clinical features and risk factors of plastic bronchitis in Mycoplasma pneumoniae pneumonia children with pulmonary consolidation: a prospective cohort study

**DOI:** 10.3389/fped.2026.1830700

**Published:** 2026-05-15

**Authors:** Yanjuan Yu, Fang Wang, Huaili Wang

**Affiliations:** 1 Department of Pediatrics, The First Affiliated Hospital of Zhengzhou University, Zhengzhou, China; 2 Respiratory Department, Children’s Hospital Affiliated to Zhengzhou University, Henan Children’s Hospital, Zhengzhou Children’s Hospital, Zhengzhou, China

**Keywords:** children, mycoplasma pneumoniae pneumonia, plastic bronchitis, pulmonary consolidation, risk factors

## Abstract

**Objective:**

To explore the clinical features and predictive factors of plastic bronchitis (PB) in children with Mycoplasma pneumoniae pneumonia (MPP) and pulmonary consolidation.

**Methods:**

Prospective observational study. Children with MPP and lung consolidation who were hospitalized and underwent bronchoscopy and treatment between January 1, 2024, and December 31, 2025, were enrolled as the study subjects. These subjects were divided into a PB group and a non-PB group. Demographic data, laboratory test results, and imaging findings were statistically analyzed to describe their clinical characteristics. Multivariate logistic regression analysis was performed to identify significant risk factors for PB in MPP children with pulmonary consolidation.

**Results:**

A total of 65 PB patients and 115 non-PB patients were enrolled. The PB group showed higher rates of respiratory distress, longer hospital stays, longer fever durations, and elevated levels of neutrophil percentage, CRP, PCT, IL-6, LDH, SF, D-dimer, and ALT. Multivariate analysis identified fever duration (OR 6.777), D-dimer (OR 1.020), and LDH (OR 1.643) as significant predictors. Bootstrap validation demonstrated good model stability (corrected AUC 0.971, shrinkage factor 0.866). Optimal cutoffs were ≥7.5 days for fever duration, >0.94 μg/mL for D-dimer, and >400.5 U/L for LDH.

**Conclusion:**

Fever duration ≥7.5 days, D-dimer >0.94 μg/mL, and LDH >400.5 U/L are independently associated with PB in MPP children with pulmonary consolidation. These associations support a link between hypercoagulability, excessive inflammation, and PB. Further studies are needed to evaluate whether early anti-inflammatory therapy or bronchoscopic intervention improves clinical outcomes.

## 1 Introduction

Mycoplasma pneumoniae pneumonia (MPP) is one of the most common types of community-acquired pneumonia (CAP) in children, particularly among school-aged children (1). In 2023, a sharp increase in Mycoplasma pneumoniae (MP) infections in China placed a significant burden on the healthcare system (2, 3). Among the radiological manifestations of MPP, pulmonary consolidation is a more severe form prone to complications (4), and plastic bronchitis (PB) is one of the most common complications of MPP with pulmonary consolidation.

Pulmonary consolidation is also one of the most common indications for bronchoscopy and therapeutic intervention in clinical practice. While some children have only minor mucus secretion, others present with massive, tree-like plugs that obstruct the airway (5). Delayed recognition can lead to respiratory distress, acute respiratory distress syndrome, or long-term sequelae such as bronchiectasis and obliterative bronchiolitis (6). Timely bronchoscopy and treatment can improve the prognosis (7).

However, it is challenging to ascertain bronchial mucus plugs or PB based solely on the findings of chest computed tomography (CT). Clinical symptoms and signs are also nonspecific, leading to frequent delays in bronchoscopy and treatment. Therefore, it is particularly important to use key clinical variables to predict whether children with pulmonary consolidation caused by MPP will develop PB. Previous studies have preliminarily explored the risk factors for the occurrence of PB in children with MPP. Zhao et al. and Cheng et al. reported that the peak fever temperature could be used as a predictive indicator (8, 9). Ma R et al. identified D-dimer levels >1.655 mg/L and LDH >364.5 U/L as independent risk factors for PB in patients with macrolide-resistant mycoplasma pneumoniae pneumonia (MRMPP) (10). Nevertheless, most of these studies were retrospective and did not specifically focus on the pulmonary consolidation subgroup, which carries the highest PB risk. Hence, this prospective study aims to investigate the clinical features and predictive risk factors of PB in MPP children with pulmonary consolidation, facilitating early diagnosis and treatment and ultimately improving prognosis.

## 2 Methods

### 2.1 Study participants and diagnostic criteria

A consecutive cohort of children under 18 years old admitted to the Department of Respiratory Medicine at the Children's Hospital Affiliated to Zhengzhou University between January 2024 and December 2025 and fulfilling the diagnostic criteria of MPP were recruited into this study (11). The study was approved by the Ethics Committee of the Children's Hospital Affiliated to Zhengzhou University (Approval No: 2024-128-001), and written informed consent was obtained from both the participants and their parents/legal guardian (s).

The inclusion criteria are as follows: (1) Presence of fever, cough, wheezing, respiratory distress, and other respiratory symptoms; (2) Lobar/pulmonary segmental consolidation confirmed by chest CT examination, with a large hyperdense shadow or infiltrative changes in the lung parenchyma; (3) A four-fold or greater increase in MP antibody titer from the acute to the convalescent phase, and/or positive detection of MP-DNA/RNA in throat swabs/bronchoalveolar lavage fluid; (4) Patients who underwent bronchoscopy and treatment. The exclusion criteria are as follows: (1) Patients with congenital airway malformations, chronic pulmonary diseases, cardiovascular diseases, neuromuscular diseases, hematological diseases, immunodeficiency disorders, or genetic metabolic diseases; (2) Patients with incomplete clinical data; (3) Co-infection with other pathogens; (4) Patients with a disease duration exceeding two weeks.

The diagnostic criteria for PB are as follows: A bronchial-shaped object is retrieved via bronchoscopy, and the result is verified by pathological examination (12, 13). The indications for bronchoscopy are based on the Chinese Guidelines for Flexible Bronchoscopy in Children (2018 Edition) (14) and the Expert Consensus on Perioperative Management of Bronchoscopy for Mycoplasma Pneumonia in Children (15). Bronchoscopy was not performed initially but was indicated only after ≥72 h of standard therapy in cases with suspected clinical or radiological progression. Based on the results of bronchoscopy, patients were divided into the PB group and the non-PB group.

### 2.2 Clinical evaluation

Clinical data were collected from all enrolled patients, including demographic information, primary clinical symptoms (fever, cough, wheezing, and dyspnea), duration of fever, length of hospital stay, the duration between hospital admission and the performance of bronchoscopy (timing) and other relevant parameters. The laboratory tests performed on the day of the patient's admission included complete blood cell count (CBC), C-reactive protein (CRP), procalcitonin (PCT), interleukin-6 (IL-6), erythrocyte sedimentation rate (ESR), liver function tests, lactate dehydrogenase (LDH), serum ferritin (SF), and D-dimer. Chest CT scans were performed to evaluate pulmonary disease. Pathogen testing is conducted using blood and sputum cultures, MP serological testing, and polymerase chain reaction (PCR) testing.

### 2.3 Statistical analysis

Statistical analyses were performed using SPSS software (version 22.0; SPSS, Chicago, IL, USA). Normally distributed data are presented as mean ± standard deviation. Continuous variables were analyzed using independent samples *t*-tests, and categorical variables were analyzed using *χ*² tests (Yates' correction). Non-normally distributed data are presented as median (interquartile range), and statistical analysis was conducted using the Mann–Whitney *U*-test. Spearman correlation analysis was employed to examine the correlations among various clinical indicators. Logistic regression analysis was employed to evaluate the significant risk factors for MPP complicated with PB. All selected variables were entered using the forced entry method based on clinical relevance and collinearity assessment. Internal validation was performed using 500 bootstrap resamples to assess model stability, including optimism-corrected AUC, shrinkage factor, and calibration curve. Receiver operating characteristic (ROC) curve analysis was used to identify potential biomarkers for children with MPP complicated with PB. A *P* < 0.05 (two-tailed) was considered statistically significant.

## 3 Results

### 3.1 Cohort selection

A total of 351 children who met the inclusion criteria were included in this study. Among these children, 171 subjects were excluded because of incomplete medical records (*n* = 21), nephrotic syndrome (*n* = 1), inherited metabolic diseases (*n* = 1), asthma (*n* = 4), congenital heart disease (*n* = 3), immunodeficiency disorders (*n* = 2), or mixed infections (*n* = 139). Ultimately, a total of 180 subjects (102 males and 78 females) were included in the analysis, comprising 65 PB cases and 115 non-PB cases. The comparison of clinical characteristics, laboratory test results and imaging findings between the two groups of patients is shown in Table 1.

**Table 1:** Clinical features of *Mycoplasma pneumoniae* pneumonia patients presented to children's hospital affiliated to Zhengzhou university.

Variables	PB (*n* = 65)	Non-PB (*n* = 115)	*p* value
Age, y	7.1 ± 2.1	7.1 ± 2.5	0.870
Sex, male/female	40/25	62/53	0.380
Hospitalization duration, day	13.0 (10.0, 15.0)	7.0 (6.0, 8.0)	**<0**.**001**
Timing, day	3.0 (3.0, 3.0)	3.0 (3.0, 3.0)	0.635
Clinical manifestations			
Fever, *n* (%)	65 (100%)	115 (100.0%)	NA
Tmax, ℃	39.8 (39.5, 40.0)	39.5 (39.2, 40.0)	0.0674
Fever duration, day	10.0 (8.0, 12.0)	6.0 (5.0, 7.0)	**<0**.**001**
Cough, *n* (%)	65 (100%)	115 (100%)	NA
Wheezing, n(%)	5 (7.7%)	4 (3.5%)	0.287
Dyspnea, n(%)	18 (27.7%)	14 (12.2%)	**<0**.**001**
Laboratory parameters			
WBC, ×10^9^/L	8.0 (6.2, 10.7)	8.4 (6.7, 10.1)	0.539
Neutrophil percentage, %	75.8 (67.5, 83.2)	75.8 (67.5, 83.2)	**<0**.**001**
PLT, ×10^9^/L	246.0 (197.0, 313.5)	257.0 (211.0, 309.0)	0.451
CRP, mg/L	43.2 (21.44, 91.8)	10.4 (5.2, 17.8)	**<0**.**001**
ESR, mm/h	33.0 (23.5, 47.0)	35.0 (24.0, 46.0)	0.567
PCT, ng/mL	0.2 (0.1, 0.4)	0.08 (0.05, 0.14)	**0**.**001**
IL-6, pg/mL	21.9 (7.1, 44.1)	10.6 (3.9, 24.0)	**0**.**0026**
LDH, U/L	568.0 (463.5, 726.0)	291.0 (261.0, 329.0)	**<0**.**001**
SF, U/L	229.5 (133.2, 508.9)	113.6 (80.0, 147.2)	**<0**.**001**
D-dimer, μg/mL	1.8 (1.1, 3.1)	0.4 (0.6, 0.8)	**<0**.**001**
ALT, U/L	34.1 (15.3, 83.1)	13.9 (10.7, 17.8)	**<0**.**001**
ALB, g/L	36.0 (32.5, 39.9)	42.1 (40.4, 44.2)	**<0**.**001**
Co-infection, n(%)	38 (58.5%)	51 (44.3%)	0.088
Chest CT imaging			
High-density consolidation, *n* (%)	65 (100%)	115 (100.0%)	NA
Pleural effusion, *n* (%)	17 (26.2%)	18 (15.7%)	0.116

The bolded *P* values indicate that there is a statistical difference.

WBC, White blood cell; PLT, Platelet; CRP, C-reactive protein; ESR, Erythrocyte sedimentation rate; PCT, Procalcitonin; IL-6, Interleukin-6; LDH, Lactate dehydrogenase; SF, Serum ferritin; ALT, Alanine Aminotransferase; ALB, Albumin.

### 3.2 Demographic and clinical characteristics

There were no statistically significant differences between the PB group and the non-PB group in terms of age, sex and timing distribution (*P* > 0.05). Fever and cough were present in all children in both groups. The PB group exhibited significantly longer fever duration, a higher incidence of respiratory distress, and longer hospital stay compared to the non-PB group. No significant differences were observed between the two groups in terms of peak fever temperature or the incidence of wheezing (*P* > 0.05).

### 3.3 Laboratory and imaging findings

Inflammatory markers, including neutrophil percentage, CRP, PCT, IL-6, LDH, SF, D-dimer, and alanine aminotransferase (ALT) were significantly elevated in the PB group, while albumin (ALB) was significantly reduced in PB patients. However, there were no significant differences in WBC, PLT, and ESR between the two groups (*P* > 0.05). Chest CT scans of all children revealed varying degrees of pulmonary consolidation, but no significant difference was observed in the incidence of pleural effusion between the two groups (*P* > 0.05).

### 3.4 Treatment and prognosis

Initial treatment was uniformly administered with first-line antibiotics-azithromycin. If fever persisted or radiological findings deteriorated after 5 days of macrolide antibiotic therapy, second-line antibiotics (doxycycline or levofloxacin) were substituted. All patients in both groups received a standard doses of intravenous methylprednisolone as anti-inflammatory therapy. There was a significant difference in hospitalization duration between the two groups (*P* < 0.001). Two children in the PB group required mechanical ventilation due to acute respiratory failure. All children improved or were cured, with no mortality cases reported.

### 3.5 Risk factors for PB

**Figure 1:**
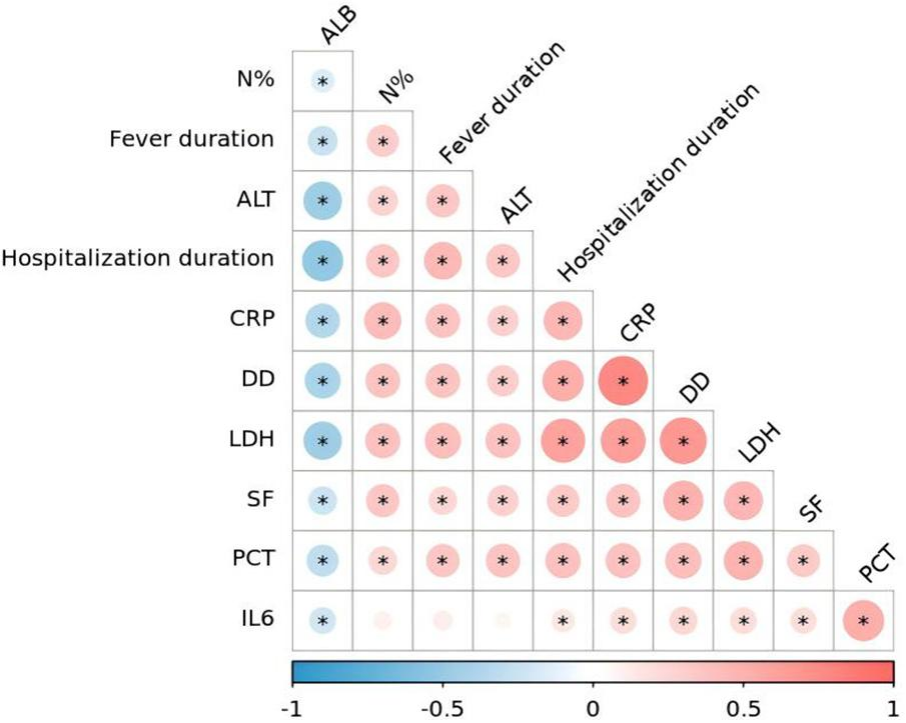
The results of the spearman correlation coefficient for the patient indicators.

Before conducting the multivariate logistic regression analysis, we performed a Spearman correlation analysis on the continuous variables (hospitalization duration, fever duration, N% CRP, PCT, IL-6, LDH, SF, D-dimer, ALT, and ALB) that showed significant differences between the two groups in the univariate analysis. The results show significant correlations among most variables, suggesting potential multicollinearity among them (Figure 1). To further evaluate multicollinearity, we constructed a pre-regression model that included all single-factor significant variables, as well as age and sex, and calculated the variance inflation factor (VIF). The results showed that the VIF for LDH was 5.11 and that for DD was 5.43 (both exceeding 5), indicating substantial multicollinearity.

**Figure 2:**
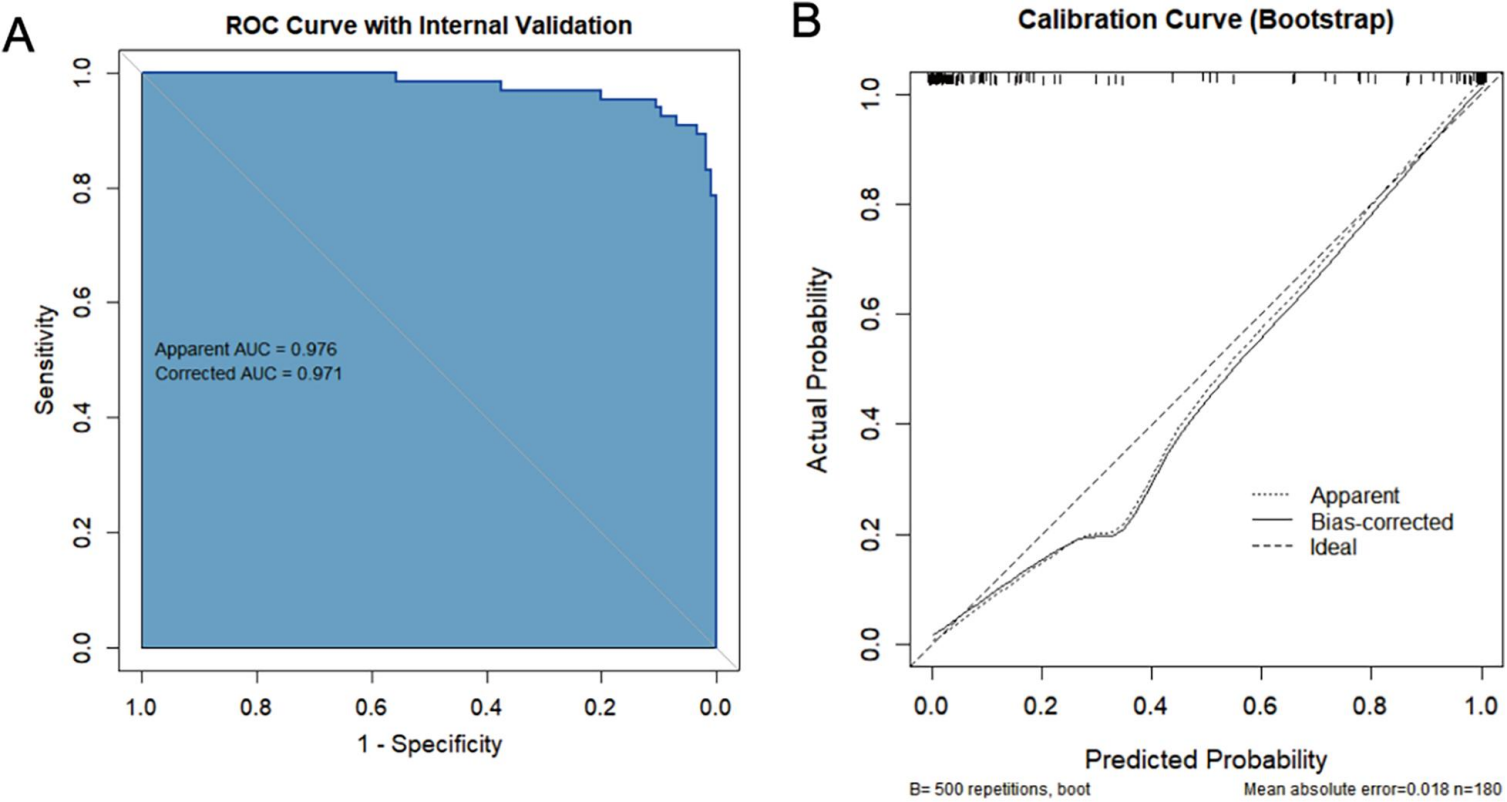
The bootstrap analysis results of the logistic model. **(A)** The ROC curve results of the Logistic regression model; **(B)** The results of the Bootstrap correction curve for the Logistic regression model.

To avoid model instability caused by collinearity and considering the relatively limited number of events, we adopted the following variable screening strategy: First, based on the clinicopathological mechanisms, candidate variables were divided into three functional clusters—persistent inflammation (duration of fever, CRP, PCT, IL-6), lung injury/airway mucus hypersecretion (LDH, SF), and hypercoagulable state (D-dimer). In each cluster, select the variable that is most directly related to clinical practice and has the strongest support from the literature as the representative. Finally, we determined three variables (duration of fever, LDH, and D-dimer) to be included in the Logistic regression model, and adjusted for age and gender. The results showed D-dimer, LDH, and fever duration were the significant predictors of PB, with odds ratios of 6.777, 1.020, and 1.643, respectively (Table 2). Bootstrap internal validation (500 resamples) showed an original AUC of 0.976 and an optimism-corrected AUC of 0.971, with a shrinkage factor of 0.866 and a calibration mean absolute error (MAE) of 0.017, indicating good model stability and minimal overfitting (Figures 2A,B).

**Table 2:** Risk factors associated with PB.

Variable	Category	*p*	Odds Ratios	95%CI	
Age	Per year increase	0.915	0.982	0.709	1.361
Sex	Male (vs. female)	0.322	0.489	0.119	2.015
D-dimer	Per 1 μg/mL increase	**0.014**	6.777	1.483	30.975
LDH	Per 1 U/L increase	**<0**.**001**	1.020	1.011	1.029
Fever duration	Per 1 day increase	**<0**.**001**	1.643	1.262	2.145

The bolded *P* values indicate that there is a statistical difference.

LDH, Lactate dehydrogenase.

**Figure 3:**
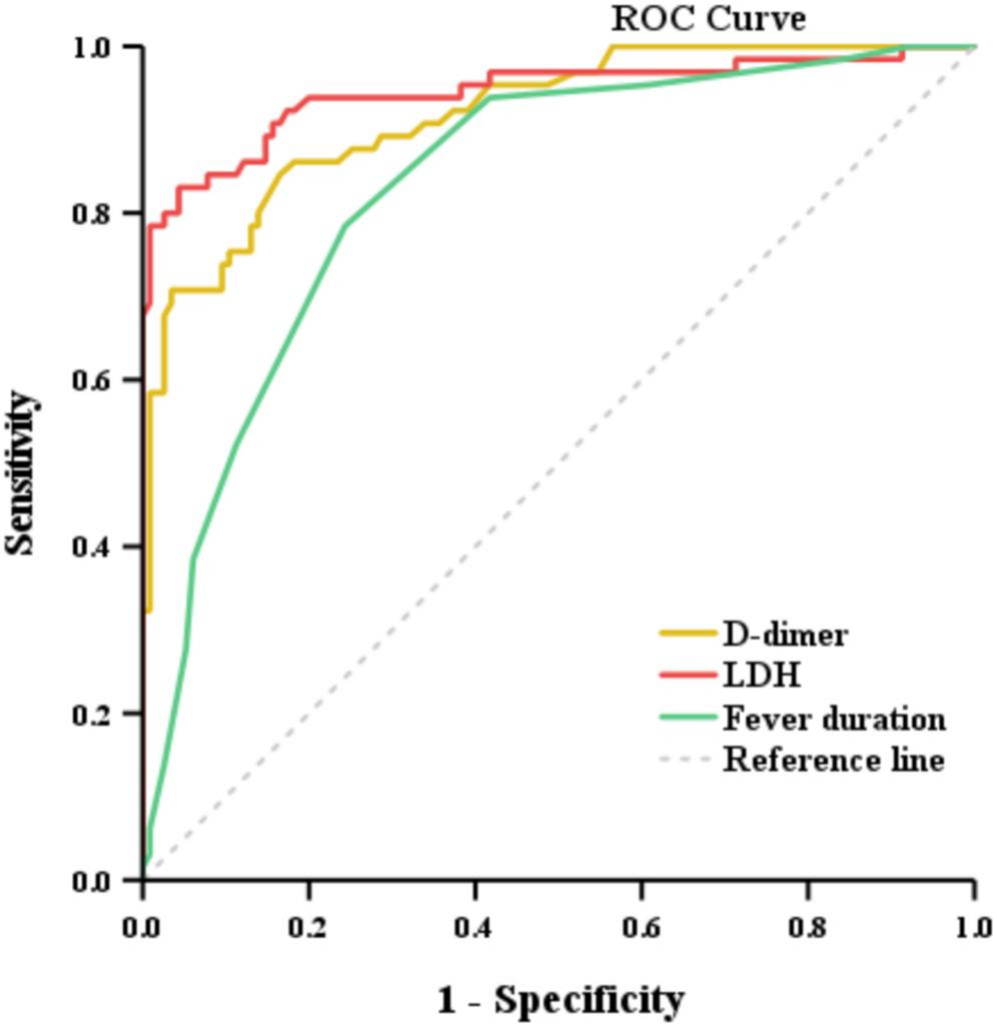
ROC curve for predictive values of the independent correlation factors of MPP with PB.

We further applied ROC curve analysis to identify predictive factors for PB, and the optimal cutoff values for sensitivity and specificity are presented in Figure 3. ROC analysis revealed that the optimal cutoff values of D-dimer, LDH, and fever duration for predicting PB were 0.94 µg/mL, 400.5 U/L, and ≥7.5 days, respectively. The corresponding sensitivity and specificity were as follows: for D-dimer were 0.846 and 0.835, for LDH were 0.831 and 0.957, and for fever duration (≥7.5 days) were 0.785 and 0.757 (Table 3).

**Table 3:** Predictive value of independent risk factors for PB.

Independent Factors	Cutoff Value	Sensitivity	Specificity	AUC	*p*
D-dimer, μg/mL	0.94	0.846	0.835	0.918	<0.001
LDH, U/L	400.5	0.831	0.957	0.945	<0.001
Fever duration, day	7.5	0.785	0.757	0.833	<0.001

LDH, Lactate dehydrogenase.

### 3.6 Value of serum D-dimer, LDH and fever duration in the diagnosis of PB in MPP

Using ROC-derived cutoff values, the four predictive factors were dichotomized into high- and low-expression groups for analysis of their associations with PB and non-PB. Chi-square tests revealed significant positive associations between PB and elevated levels of D-dimer (*P* < 0.001), LDH (*P* < 0.001), and fever duration (*P* < 0.001) (Table 4).

**Table 4:** PPV and NPV of independent factors with PB in MPP children.

Independent Factors	Total	PB	Non-PB	*χ* ^2^	*P*	PPV	NPV
D-dimer, n(%)							
≥0.94 μg/mL	74 (41.1)	55 (84.6)	19 (16.5)	79.535	<0.001	0.743	0.906
<0.94 μg/mL	106 (58.9)	10 (15.4)	96 (83.5)				
LDH, *n* (%)							
≥400.5 U/L	59 (32.8)	54 (83.1)	5 (4.3)	116.820	<0.001	0.915	0.909
<400.5 U/L	121 (67.2)	11 (16.9)	110 (95.7)				
Fever duration, n(%)							
≥7.5 day	79 (43.9)	51 (78.5)	28 (24.3)	49.380	<0.001	0.646	0.861
<7.5 day	101 (56.1)	14 (21.5)	87 (75.7)				

PPV, Positive predictive value; NPV, Negative predictive value.

When the serum D-dimer level exceeded 0.94 µg/mL, the positive predictive value (PPV) was 74.3%, and the negative predictive value (NPV) was 90.6%. For serum LDH levels above 400.5 U/L, the PPV was 91.5%, and the NPV was 90.9%. Similarly, for a fever duration longer than 7.5 days, the PPV was 64.6% and the NPV was 86.1% (Table 4).

## 4 Discussion

In recent years, with the widespread use of bronchoscopy, MPP-associated PB has attracted increasing attention (12, 16, 17). The pathogenesis of PB remains unclear; previous studies have suggested that ciliary motility dysfunction, excessive inflammatory response, and abnormal mucus secretion following MP infection are the primary mechanisms underlying MPP complicated by PB (18–20). Pediatric MPP complicated by PB lacks specific clinical manifestations and imaging indicators. Delayed diagnosis and treatment may lead to severe consequences. Therefore, identifying risk factors for PB in children with MPP is particularly important.

In this study, all patients presented with fever and cough. There were no significant differences between the two groups in peak fever temperature (*P* = 0.0674) or pleural effusion incidence (26.2% vs. 15.7%, *P* = 0.116). This suggests that the duration, rather than the peak intensity, of inflammation drives PB formation, as prolonged fever promotes airway secretion concentration and mucociliary impairment. Meanwhile, pleural effusion reflects pleural inflammation, whereas PB involves endobronchial casts; thus, the absence of pleural effusion does not rule out PB. The incidence of dyspnea in the PB group was significantly higher than that in the non-PB group, suggesting that dyspnea in children with MPP presenting radiologically as pulmonary consolidation may be associated with a higher likelihood of PB. In this study, the fever duration in the PB group was significantly longer than that in the non-PB group, which is consistent with previous studies (21–23), suggesting that children with PB may exhibit a more severe inflammatory response. Sustained inflammatory responses may lead to airway edema and narrowing, potentially impeding the timely clearance of airway secretions. Concurrently, during a high fever, insensible water loss from the airway increases, further elevating the risk of bronchoplastic formation (24, 25). Zhao and Cheng reported that peak body temperature could serve as an independent risk factor for PB (8, 9). However, in contrast to their findings, our study revealed no significant difference in peak fever temperature between the two groups. Instead, a fever duration of ≥7.5 days emerged as an independent risk factor for predicting PB in MPP patients, exhibiting high sensitivity (78.5%) and specificity (75.7%), which is consistent with the findings of Hao et al.'s research (26). Compared to single-point temperature measurements, fever duration better reflects the systemic inflammatory and disease severity. Vigilance is also required for PB in MPP children who experience prolonged high fever.

In this study, the inflammatory markers including neutrophil percentage, CRP, PCT, IL-6, LDH, SF and the coagulation marker D-dimer were significantly higher in the PB group compared to the non-PB group. Lin et al. (27) also observed that MPP complicated by PB elevated levels of neutrophils, CRP, PCT, D-dimer, LDH, and AST compared to those in patients without PB. This is consistent with our findings, suggesting that excessive inflammatory responses and coagulation abnormalities may be involved in the pathogenesis of PB. D-dimer and LDH levels can serve as indicators of disease severity in MPP (28, 29). In this study, multivariate analysis identified D-dimer and LDH as independent predictors of PB in children with MPP. Yue et al. reported that D-dimer serves as a predictor of PB or necrotizing pneumonia in children with MPP (30). Niu et al. identified D-dimer levels >1.655 mg/L and LDH >364.5 U/L as independent risk factors for the PB in patients with macrolide-resistant mycoplasma pneumoniae pneumonia (MRMPP) (10). Our study demonstrates that a D-dimer level >0.94 μg/mL and an LDH >400.5 U/L are significant predictive factors for PB in MPP. Systemic inflammation caused by MP infection has been reported to induce an imbalance between coagulation and anticoagulation systems (31). Such an imbalance, if present, could theoretically reduce gas exchange and alter microcirculation, potentially contributing to inflammatory exudation and sputum retention. However, whether these changes directly cause PB formation remains unclear based on our observational data. Therefore, monitoring serum LDH and D-dimer levels may serve as important early warning indicators for the development of PB in children with MPP. For such children, clinicians should maintain heightened vigilance, enhance disease monitoring and assessment, and consider performing bronchoscopy as soon as clinically indicated.

Furthermore, in this study, ALT levels in the PB group were higher than those in the non-PB group, while ALB levels were significantly lower. On one hand, MP infection can impair liver function and reduce hepatic synthesis of albumin. On the other hand, MP interacts with airway epithelial cells, leading to activation of inflammasomes and triggering an overactive immune-inflammatory response. This increases capillary permeability and leads to albumin leakage. Hypoalbuminemia subsequently promotes fluid leakage into the trachea. Under the influence of airway inflammatory factors, plasma exudate and fibrin accumulate, undergo degenerate, and necrotize within the airways, and ultimately form plugs (32). Therefore, decreased serum ALB levels are closely associated with the development of PB in MPP.

In recent years, the number of PB patients caused by MP infection has gradually increased (3). In terms of treatment, bronchoscopic extraction or removal of bronchial casts is an important method to improve ventilation in children with PB (33). Of course, anti-mycoplasma therapy and corticosteroid-based anti-inflammatory therapy are equally important. Previous studies have reported that treatment with heparin and methylprednisolone can shorten the duration of illness and reduce hospital length of stay (34, 35). However, the impact of anticoagulant and anti-inflammatory therapies on the formation and prognosis of PB requires further investigation. Macrolide antibiotics are the first-line treatment for pediatric MPP. For MRMPP, refractory mycoplasma pneumoniae pneumonia (RMPP), or Severe Mycoplasma Pneumonia (SMPP), second-line anti-mycoplasma agents such as tetracyclines or fluoroquinolone antibiotics may be effective (36). In this study, bronchoscopic lavage and combined anti-mycoplasma and anti-inflammatory therapy, all patients showed improvement or were cured, with no mortality cases, indicating an overall favorable prognosis.

This study has several limitations. It is crucial to note that our analyses were performed on a cohort defined by clinical need for bronchoscopy. This selection criterion inherently enriches for moderate-to-severe cases, which, while providing a powerful dataset to investigate drivers of PB, limits the generalizability of our prevalence estimates to the broader, milder MPP spectrum. Consequently, the observed ORs and cutoff values may be overestimated relative to an unselected MPP population, and external validation is needed. Furthermore, we did not analyze the long-term follow-up outcomes of children in the PB group, which prevents a comprehensive assessment of the long-term prognosis of PB. Therefore, extending the follow-up period is crucial for revealing the long-term impact of PB. Additionally, treatment modifications may have affected secondary outcomes (fever duration, Hospitalization duration, etc.) but not baseline PB predictors, as all predictive variables were collected on admission.

In conclusion, our study identified that fever duration ≥7.5 days, D-dimer >0.94 µg/mL, and LDH >400.5 U/L serve as independent predictors for PB in MPP children with pulmonary consolidation.

## Data Availability

The raw data supporting the conclusions of this article will be made available by the authors, without undue reservation.

## References

[1] Li L Ma J Guo P Song X Li M Yu Z Molecular beacon based real-time PCR p1 gene genotyping, macrolide resistance mutation detection and clinical characteristics analysis of Mycoplasma pneumoniae infections in children. BMC Infect Dis. (2022) 22(1):724. 10.1186/s12879-022-07715-6 36068499 PMC9447981

[2] Hao C Li Z Yu Y Wang S Wang G Jia G The high-risk CCL14 lineage drives severe Mycoplasma pneumoniae pneumonia in children: a study in central China. Clin Exp Pediatr. (2026). 10.3345/cep.2025.02796 41986956 PMC13244062

[3] Li Z Hao C Jia G Liang Q Wang Q Wu Y Multi-omics analysis of host airway responses in pediatric Mycoplasma pneumoniae pneumonia reveals potential mechanisms of disease exacerbation caused by co-infection. NPJ Biofilms Microbiomes. (2025) 11(1):230. 10.1038/s41522-025-00859-8 41413425 PMC12714720

[4] Huang X Gu H Wu R Chen L Lv T Jiang X Chest imaging classification in Mycoplasma pneumoniae pneumonia is associated with its clinical features and outcomes. Respir Med. (2024) 221:107480. 10.1016/j.rmed.2023.107480 38043865

[5] Jiang YQ Tian JM Cheng FF Kong XX Shi T Bian YX Volume ratio of pulmonary lesions as a novel indicator for predicting the occurrence of plastic bronchitis in children with Mycoplasma pneumoniae pneumonia. Eur J Pediatr. (2025) 184(7):409. 10.1007/s00431-025-06251-0 40490539

[6] Huang F Gu W Diwu J Zhang X He Y Zhang Y Etiology and clinical features of infection-associated plastic bronchitis in children. BMC Infect Dis. (2023) 23(1):588. 10.1186/s12879-023-08529-w 37679703 PMC10486060

[7] Zhong H Yin R Zhao R Jiang K Sun C Dong X. Analysis of clinical characteristics and risk factors of plastic bronchitis in children with Mycoplasma pneumoniae pneumonia. Front Pediatr. (2021) 9:735093. 10.3389/fped.2021.735093 34733807 PMC8558491

[8] Zhao L Zhang T Cui X Zhao L Zheng J Ning J Development and validation of a nomogram to predict plastic bronchitis in children with refractory Mycoplasma pneumoniae pneumonia. BMC Pulm Med. (2022) 22(1):253. 10.1186/s12890-022-02047-2 35761218 PMC9235233

[9] Cheng S Lin J Zheng X Yan L Zhang Y Zeng Q Development and validation of a simple-to-use nomogram for predicting refractory Mycoplasma pneumoniae pneumonia in children. Pediatr Pulmonol. (2020) 55(4):968–74. 10.1002/ppul.24684 32040888

[10] Ma R Bai T Yuan B Zhang L Li S Ma L Risk factor analysis of plastic bronchitis among 126 children with macrolide-resistant Mycoplasma pneumoniae pneumonia with mutations at the A2063G site after bronchoscopy examination: a nomogram prediction model. Front Pediatr. (2025) 13:1521954. 10.3389/fped.2025.1521954 40083439 PMC11903698

[11] Subspecialty Group of Respiratory, the Society of Pediatrics, Chinese Medical Association, China National Clinical Research Center of Respiratory Diseases, Editorial Board, Chinese Journal of Pediatrics. Evidence based guideline for the diagnosis and treatment of Mycoplasma pneumoniae pneumonia in children (2023). Pediatr Investig. (2025) 9(1):1–11. 10.1002/ped4.12469 PMC11998179 40241891

[12] Mu SY Zou YX Guo YS Huang B Gao WW Zhang T Clinical characteristics and predictive factors for plastic bronchitis in children with severe Mycoplasma pneumoniae pneumonia. Zhonghua Er Ke Za Zhi. (2024) 62(9):861–6. 10.3760/cma.j.cn112140-20240417-00272 39192444

[13] Seear M Hui H Magee F Bohn D Cutz E. Bronchial casts in children: a proposed classification based on nine cases and a review of the literature. Am J Respir Crit Care Med. (1997) 155(1):364–70. 10.1164/ajrccm.155.1.9001337 9001337

[14] Experts Group of Pediatric Respiratory Endoscopy TESCoNHCEC, Pediatric Section of Chinese Medical Doctor Association; Pediatric Respiratory Endoscopy Committee, Endoscopists Section of Chinese Medical Doctor Association; Pediatric Interventional Respirology Group, Maternal and Pediatric Minimally Invasive Section of Chinese Maternal and Child Health Association; Bronchoscopy Collaboration Subgroup of Respirology Group. Pediatric Section of Chinese Medical Association Less. Guideline of pediatric flexible bronchoscopy in China (2018). Chin J Appl Clin Pediatr. (2018) 33(13):983–9. 10.3760/cma.j.issn.2095-428X.2018.13.006

[15] Pediatric Respiratory Standardization Diagnosis and Treatment and Quality Control Working Committee. China Quality Association for Pharmaceuticals. Expert consensus on the perioperative management of bronchoscopy for Mycoplasma pneumoniae pneumonia in children in China. Chin J Pract Pediatr. (2024) 39(12):881–6. 10.19538/j.ek2024120601

[16] Yang L Zhang Y Shen C Lu Z Hou T Niu F Clinical features and risk factors of plastic bronchitis caused by Mycoplasma pneumoniae pneumonia in children. BMC Pulm Med. (2023) 23(1):468. 10.1186/s12890-023-02766-0 37996853 PMC10668422

[17] Zhang H Yang J Zhao W Zhou J He S Shang Y Clinical features and risk factors of plastic bronchitis caused by refractory Mycoplasma pneumoniae pneumonia in children: a practical nomogram prediction model. Eur J Pediatr. (2023) 182(3):1239–49. 10.1007/s00431-022-04761-9 36633659 PMC10023623

[18] Hu J Ye Y Chen X Xiong L Xie W Liu P. Insight into the pathogenic mechanism of Mycoplasma pneumoniae. Curr Microbiol. (2022) 80(1):14. 10.1007/s00284-022-03103-0 36459213 PMC9716528

[19] Hao Y Kuang Z Jing J Miao J Mei LY Lee RJ Mycoplasma pneumoniae modulates STAT3-STAT6/EGFR-FOXA2 signaling to induce overexpression of airway mucins. Infect Immun. (2014) 82(12):5246–55. 10.1128/IAI.01989-14 25287927 PMC4249270

[20] Meldrum OW Chotirmall SH. Mucus, microbiomes and pulmonary disease. Biomedicines. (2021) 9(6):675. 10.3390/biomedicines9060675 34199312 PMC8232003

[21] Xu JX Li J Zhou R Xia Q Zhang Z Xuan AL Clinical characteristics and predictive models of plastic bronchitis caused by mycoplasma pneumoniae pneumonia in children. J Formos Med Assoc. (2026) 125(1):37–43. 10.1016/j.jfma.2025.07.012 40645867

[22] Dong JM Zhou CQ Zhen YY. Risk factor analysis of Mycoplasma pneumoniae pneumonia complicated with plastic bronchitis in children: a single-center retrospective study. Clin Respir J. (2025) 19(12):e70142. 10.1111/crj.70142 41344998 PMC12678006

[23] Zheng C Zheng Z Hu X Huang Y Han Y. Risk analysis of poor prognosis and follow-up observation of children with Mycoplasma pneumoniae pneumonia complicated by plastic bronchitis. Front Pediatr. (2025) 13:1565773. 10.3389/fped.2025.1565773 40656192 PMC12245785

[24] Ma Y Gu Y Zhang X Gu W Wang T Sun H High expression of MUC5AC, MUC5B, and layilin plays an essential role in prediction in the development of plastic bronchitis caused by MPP. Front Microbiol. (2022) 13:911228. 10.3389/fmicb.2022.911228 35770160 PMC9234514

[25] Lee YC Chang CH Lee WJ Liu TY Tsai CM Tsai TA Altered chemokine profile in refractory Mycoplasma pneumoniae pneumonia infected children. J Microbiol Immunol Infect. (2021) 54(4):673–9. 10.1016/j.jmii.2020.03.030 32299786

[26] Dong H Zhang Y Yan LM Zhang HY Zhang L. Clinical characteristics and risk factor analysis of children with severe Mycoplasma pneumoniae pneumonia complicated by plastic bronchitis. Transl Pediatr. (2025) 14(10):2561–71. 10.21037/tp-2025-438 41216456 PMC12597197

[27] Li L Wang D Yang R Liao X Wu L. Application of decision tree model in diagnosis of mycoplasma pneumoniae pneumonia with plastic bronchitis. Ital J Pediatr. (2025) 51(1):95. 10.1186/s13052-025-01934-8 40128882 PMC11934725

[28] Lee E Choi I. Clinical usefulness of Serum lactate dehydrogenase levels in Mycoplasma pneumoniae pneumonia in children. Indian J Pediatr. (2022) 89(10):1003–9. 10.1007/s12098-022-04205-0 35665905

[29] Zheng Y Hua L Zhao Q Li M Huang M Zhou Y The level of D-dimer is positively correlated with the severity of Mycoplasma pneumoniae pneumonia in children. Front Cell Infect Microbiol. (2021) 11:687391. 10.3389/fcimb.2021.687391 34336714 PMC8319762

[30] Yue Y Lian T Kang L Liu S Geng W Xu M. D-dimer serves as predictor of plastic bronchitis or necrotizing pneumonia in children with Mycoplasma pneumoniae pneumonia. Front Pediatr. (2025) 13:1604253. 10.3389/fped.2025.1604253 40735605 PMC12303902

[31] Shen F Dong C Zhang T Yu C Jiang K Xu Y Development of a nomogram for predicting refractory Mycoplasma pneumoniae pneumonia in children. Front Pediatr. (2022) 10:813614. 10.3389/fped.2022.813614 35281240 PMC8916609

[32] Soeters PB Wolfe RR Shenkin A. Hypoalbuminemia: pathogenesis and clinical significance. JPEN J Parenter Enteral Nutr. (2019) 43(2):181–93. 10.1002/jpen.1451 30288759 PMC7379941

[33] Yuan L Zhuo ZQ Zhu QG Li MZ Wu XD. Effect of bronchoscopy on plastic bronchitis in children. Pediatr Pulmonol. (2024) 59(11):2984–6. 10.1002/ppul.27115 39031864

[34] Zheng B Zhao J Cao L. The clinical characteristics and risk factors for necrotizing pneumonia caused by Mycoplasma pneumoniae in children. BMC Infect Dis. (2020) 20(1):391. 10.1186/s12879-020-05110-7 32487034 PMC7268704

[35] You SY Jwa HJ Yang EA Kil HR Lee JH. Effects of methylprednisolone pulse therapy on refractory Mycoplasma pneumoniae pneumonia in children. Allergy Asthma Immunol Res. (2014) 6(1):22–6. 10.4168/aair.2014.6.1.22 24404389 PMC3881395

[36] Yu Y Jin X Zhang X Shen Y. Pulmonary thrombotic complication of Mycoplasma pneumoniae pneumonia in Chinese children: clinical feature and risk factor analysis. Pediatr Infect Dis J. (2024) 43(6):505–10. 10.1097/INF.0000000000004287 38359345

